# Dissipation-Dependent Thermal Escape from a Potential Well

**DOI:** 10.3390/e23101315

**Published:** 2021-10-09

**Authors:** Chungho Cheng, Matteo Cirillo, Niels Grønbech-Jensen

**Affiliations:** 1 Department of Mechanical and Aerospace Engineering, University of California, Davis, CA 95616, USA; argcheng@ucdavis.edu (C.C.); ngjensen@ucdavis.edu (N.G.-J.); 2 Dipartimento di Fisica and MINAS Lab, Università di Roma “Tor Vergata”, 00133 Roma, Italy; 3 Department of Mathematics, University of California, Davis, CA 95616, USA

**Keywords:** macroscopic quantum coherence, josephson effect, superconductive tunnelling

## Abstract

Langevin simulations are conducted to investigate the Josephson escape statistics over a large set of parameter values for damping and temperature. The results are compared to both Kramers and Büttiker–Harris–Landauer (BHL) models, and good agreement is found with the Kramers model for high to moderate damping, while the BHL model provides further good agreement down to lower damping values. However, for extremely low damping, even the BHL model fails to reproduce the progression of the escape statistics. In order to explain this discrepancy, we develop a new model which shows that the bias sweep effectively cools the system below the thermodynamic value as the potential well broadens due to the increasing bias. A simple expression for the temperature is derived, and the model is validated against direct Langevin simulations for extremely low damping values.

## 1. Introduction

Thermally activated escape from a potential energy well is a topic of ubiquitous interest in condensed matter physics, and the quasi-equilibrium of the metastable state prior to the escape is of course crucially important for understanding the escape process. The pioneering work of Kramers [1] analyzed the problem for relatively low temperature and relatively high damping such that one can assume near-equilibrium conditions. This analysis has produced a tremendously powerful tool for a broad class of problems. Notably, Kramers’ theory has, over the past several decades, become critically important for the analysis of the finite Josephson potential [2] and its characteristics. While the features of the potential are not directly observable, they can be illuminated by the statistics of measurable transitions from a zero-voltage state through a bias sweep that progressively tilts the potential and thereby lowers the potential barrier until the system makes the transition [3]. Thus, from an ensemble (an ideal gas) of such bias sweep experiments, it is, within the assumptions of the theory, possible to assess self-consistency with the expectations of an assumed functional form for the potential.

Due to the irreversibility of the escape, the bias sweep, which shifts the potential well from deep to shallow, is an integral part of the interpretation of the measurable switching statistics. The departure from quasi-equilibrium was elaborated upon by Büttiker, Harris, and Landauer (BHL) [4], where the interplay between the escape time scale and the damping parameter is recognized as having a significant effect on the energy distribution prior to switching. Since it is self-evident that an escape from the well happens to a system, which has an energy of at least the energy barrier, the BHL theory outlines that the system may not have time to correctly re-equilibrate (reheat) between subsequent escapes if the damping, which sets an inverse time scale for re-equilibration, is small enough. As a result, a low-damping system will exhibit an escape distribution that reflects an effective temperature lower than the thermodynamic temperature, and the statistics will depart from that of Kramers’ theory. Although the manifestations of the mismatch between the time scale of escape and equilibration have been further investigated in, e.g., [5,6], the BHL theory has been considered much less frequently than the traditional Kramers theory as representative for the expectations in Josephson bias sweep experiments, even if the damping parameter is generally not well known in the metastable zero-voltage state. This is especially notable over the past couple of decades, during which the possibility for observing macroscopic quantum states was proposed for Josephson systems at very low temperatures [7], where the damping is also expected to be very small. At the root of demonstrating that a macroscopic quantum state has been achieved is the observation that the escape statistics from bias sweep experiments significantly departs from that of the classical expectation, thereby indicating that the anomalous escape statistics may be caused by quantum mechanical tunneling through the energy barrier in addition to the classical path over the energy barrier. Such assertion was made in [8,9], and subsequently by many others, including more recently in [10,11], and most of the interpretations of a “crossover” temperature between classical and quantum regimes have used the Kramers theory as its basis [12].

The assertion of the “crossover” from classical to quantum behavior of Josephson junctions systems has led to a bulk of literature and efforts investigating how the quantum state can be exploited as elementary bits for quantum computation [13]. Additionally, with the assumption of working with a device in the quantum regime, many dynamical experiments have been conducted to further the understanding of how to manipulate a quantum device through application of, e.g., microwaves and bias pulses that induce measurable resonant escape signatures that can be interpreted as, e.g., Rabi oscillations, Ramsey fringes, and other features akin to those found in atomic physics. For Josephson junctions, however, many of these nonequilibrium features have been directly attributed to classical resonant transients of the same driven-damped nonlinear oscillator (the resistively and capacitively shunted junction (RCSJ)) in the classical regime (see the review of [14] and, e.g., [15,16,17]). In addition, similarities between fast-sweep experiments [18], which show modulated switching distributions, and classical simulations [19,20] of a low-dissipation RCSJ model have been found. Those simulations revealed that, below a certain threshold for the ratio of the normalized damping and sweep parameters, the metastable state exhibits initial condition-induced coherent oscillations, which directly affect the subsequent escape statistics.

In light of the often ambiguous interpretations of experiments, it is the aim of this work to investigate how well the Kramers and BHL theories conform to the classical Josephson escape statistics as obtained from a Langevin description of the RCSJ model. We are especially interested in the role of the damping parameter, since this parameter is not always well known in experiments, and since both the BHL theory and the work in [19] show that this may directly influence the statistics. The remainder of the paper is structured as follows: In the next section we briefly review the main elements of the RCSJ model of Josephson junctions and review the approaches to thermal escape of Kramers [1] and BHL [4]; in Section 3, we show the results obtained from the numerical integration with “flat” initial conditions and compare those with the theoretical models and discuss the obtained results, also trying to frame those in the context of existing experimental results and parameters. In Section 4, we present a model for interpreting the presented results for the very low dissipation case. In Section 5, we have concluding remarks.

## 2. Numerical Approach and Model Equations

The *RCSJ* model for a single Josephson tunnel junction is an electrical circuit in which an element described by Josephson DC and AC equations is placed in parallel with a capacitor, a resistor, and a DC bias current. The capacitor *C* of the model gives account for the “parallel plates” structure of a tunnel junction, while the loss parameter indicates the presence of dissipative effects which could be ascribed to normal electron tunneling or to quasi-particle tunneling (below the superconducting gap of the junctions) or to other dissipative processes [2]. Referring to the same notations used in previous work [14,20] the nonlinear equation describing such a parallel, and DC-biased, combination in normalized form is

(1) $$\ddot{\varphi }+\alpha \dot{\varphi }=-sin\varphi +\eta +n\left(t\right)$$

where, in the right hand side, the first two terms represent normalized force seen as $-\frac{dU}{d\varphi }$ with U=(1−cosφ)−ηφ. The extra term *n*(*t*) on the right hand side is a noise term such that

(2) $$<n\left(\tau \right)n\left(\tau^{\prime }\right)>=2\alpha \;\theta \;\delta \left(\tau -\tau^{\prime }\right)\;\mathrm{and}\;<n\left(\tau \right)>=0$$

The variable *θ* is the normalized thermodynamic temperature of the system; i.e., the Boltzmann energy *k_B_T* (*k_B_* = 1.38 × 10^−23^ J/K) relative to the maximum Josephson energy *E_j_* = Φ_0_ *I_c_*/2π (with Φ_0_ = 2.07 × 10^−15^ Wb being the flux-quantum) and *I_c_* is the maximum critical current [2]. More terms can be added to the right hand side of Equation (1) to mimic several different experimental situations; however, for the purposes of the present work we just consider the bias term and the thermal noise.

We considered two types of initial conditions for the system (1). The first is “flat” initial data in which we just set $\varphi_{0}=\varphi \left(t=0\right)=0,\;{\dot{\varphi }}_{0}=\dot{\varphi }\left(t=0\right)=0$; this type of initial data is very reasonable for the kind of escape simulations we perform (starting at *η* = 0). However, these initial conditions do not take into consideration the initial temperature of the system, and therefore we also set up numerical integrations considering even the “bath” temperature when fixing the initial conditions. In most cases, the results generated by the two sets of initial conditions were not very different, as will be clear from the result that we present.

“Thermal” initial conditions at *t* = 0, where *η* = 0, are $\left(\varphi ,\dot{\varphi }\right)=\left(\varphi_{0},{\dot{\varphi }}_{0}\right)$, and are drawn from the Boltzmann density distribution functions such that $<\varphi_{0\;}{\dot{\varphi }}_{0}>=0$ and

(3) $$\rho \left(\varphi_{0}\right)=\frac{\exp\left(-\frac{U\left(\varphi_{0}\right)}{\theta }\right)}{\int_{-\pi }^{\pi }\;\exp\left(-\frac{U\left(\varphi_{0}\right)}{\theta }\right)d\varphi_{0}}$$

(4) $$\rho \left({\dot{\varphi }}_{0}\right)=\frac{\exp\left(-\frac{{\dot{\varphi }}_{0}^{2}}{2\theta }\right)}{\sqrt{2\pi \theta }}$$

Here, $\varphi_{0}\in \left[-\pi ,\;\pi \right)$, ${\dot{\varphi }}_{0}\in \left(-\infty ,\infty \right)$, and $U\left(\varphi_{0}\right)=1-cos\varphi_{0}-\eta \varphi_{0}$. This initial condition ensures that an ensemble of simulations will represent a thermal set of initial conditions.

It is worth noting that the physical identification/meaning of dissipation can generate problems for Josephson circuits modeling and it is somewhat difficult to determine particular damping values when junctions are current-biased in the zero voltage state for which, by nature of the superconducting state, ohmic dissipation is zero. We do not, in this work, address estimating the damping parameter. Instead, we investigate the Langevin model (1)–(2) down to very low damping values and compare the results with existing theoretical approaches, namely Kramers theory [1] and the BHL model [4], both predicting specific responses for systems described by Equation (1).

**Figure 1:**
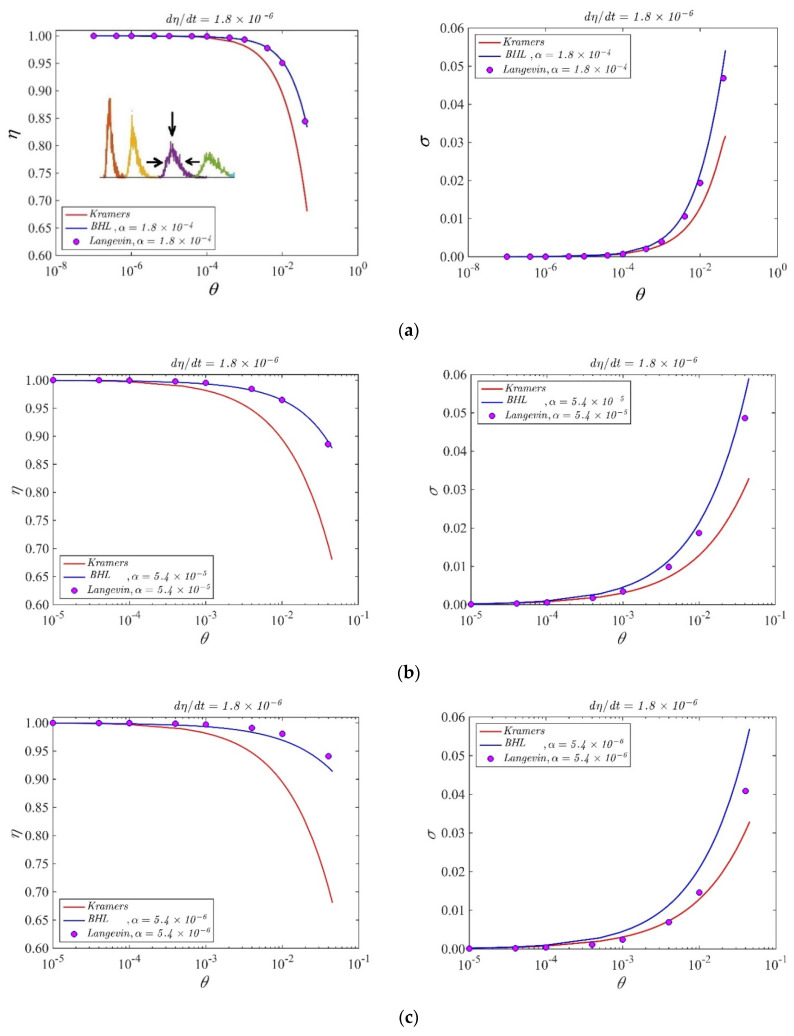
Peak position (left panels) and width (right panels) of statistical escape distributions obtained sweeping the temperature in the shown intervals for a sweep rate $\dot{\eta }$ = 1.8 × 10^−6^ for different values of the loss parameter (indicated in the panels). The dots are the results of numerical simulations, the lines BHL theory. (**a**–**c**) are relative to different values of the dissipation parameter which is indicated on the top of each panel. The inset in the left plot of (**a**) is an exemplificatory sketch for the quantities we trace: the width of the distributions is indicated by the horizontal arrows and the peak position, moving toward higher values of bias current lowering the temperature, by the vertical arrow. From right to left, the values of θ for the distributions in the inset are: 1.25 × 10^−2^, 8 × 10^−3^, 4 × 10^−3^, 2 × 10^−3^.

As the normalized sweep rate $\dot{\eta }=\frac{d\eta }{dt}$ is naturally crucial for the outcome of escape from a potential well generated by a bias sweep [18,19,20], we need to also consider this parameter in the discussion. A typical sweep simulation of Equation (1) starts at *t* = 0 with $\eta =t\dot{\eta }$ using the statistically robust stochastic Verlet algorithm [21], which is designed to give near time-step independent statistics, thus comfortably allowing for statistically correct results for a normalized time step of Δ*t* = 0.02. Signifying escape, the simulation is stopped when $\varphi >\pi -sin^{-1}\eta$, and the value of *η* is then recorded as the switching current. This procedure is repeated many times in order to generate a density distribution *P*(*η*) of these switching currents. Representative distributions are visualized in the inset of Figure 1a (left panel), where we show four distributions obtained for decreasing values of temperature (decreasing the temperature, the distributions become more peaked).

(5) $$\Gamma_{K}=\frac{\omega_{p}}{2\pi }e^{\frac{-E_{j}\Delta U}{k_{B}T}}$$

where $\Delta U=\left[\sqrt{1-\eta^{2}}-\eta cos^{-1}\eta \right]$ is the bias-dependent height of the Josephson potential barrier and the prefactor is the normalized attempt frequency $\omega_{p}=\sqrt[{4}]{1-\eta^{2}}$ (the normalized Josephson plasma frequency), measured in units of $\omega_{0}=\sqrt{\frac{2\pi I_{c}}{\Phi_{0}C}}$. Kramers also derived an expression for very low damping (containing an explicit dissipation term) which can also be seen as a limit form of the BHL model predicting the occurrence of escape through the normalized escape rate [4]:

(6) $$\Gamma_{B}=\frac{{\left[1+\left(\frac{4k_{B}T}{\alpha I_{b}}\right)\right]}^{1/2}-1}{{\left[1+\left(\frac{4k_{B}T}{\alpha I_{b}}\right)\right]}^{1/2}+1}\left[\frac{\alpha I_{b}}{k_{B}T}\right]\frac{\omega_{p}}{2\pi }e^{\frac{-E_{j}\Delta U}{k_{B}T}}$$

In our simulations, containing an explicit dissipation term, we use this latest equation in order to trace the agreement with a theoretical model. As pointed out in [4], when *α* tends to infinity, Equation (4) reduces to Equation (3); i.e., the limit of high dissipation BHL model limits Kramers’ escape rate. In Equation (4), the parameter $I_{b}=\frac{3}{10}\;16E_{j}{\left[2\left(1-\eta \right)\right]}^{5/4}$. We list in Table 1 key notations for Josephson parameters and equations for direct comparison between our present work and [4].

## 3. Results for Flat Initial Conditions

As specified above, for “flat” initial conditions we intend $\varphi \left(0\right)=0,\dot{\varphi }\left(0\right)=0$. In the panel of Figure 1 we show the results obtained from the statistical escape distributions with these initial conditions, sweeping the variable *θ* in the shown intervals for a fixed value of the sweep rate $\dot{\eta }=1.8\times 10^{-6}$. In the inset of Figure 1a we also show typical histograms obtained as results of the escape processes, where we can see the peak moving left toward higher currents and squeezing in width as a consequence of temperature decrease. We see in Figure 1a,b that the agreement between BHL model and the simulations is very good when the values of the dissipation parameter α are around 10^−4^, 10^−5^. However, in Figure 1c we see that, when the loss parameter approaches the value of the normalized sweep rate, the agreement between theory and simulations becomes less good. The significance of the parameter κ=αη˙ has been demonstrated in [19,20]. For values of the dissipation parameter of the order of 0.1, the features of the histograms (peak position and peak width) are well described by both Kramers and BHL models, as expected from the arguments of the previous section. When decreasing α below this value, only the theoretical distributions obtained from Equation (4) follow the numerical distributions. The agreement is reasonable down to *α* = 10^−6^, but below this value the numerical data no longer match the theory.

Before proceeding to describe the statistical behavior for the lowest values of dissipation, we estimate the values of experimentally relevant Josephson junction parameters. Typical normalized dissipation parameters presented in literature are in the range (0.001–0.05) [15,16,17,18,19,20]. Normalized sweep rates of published experimental results range in the interval (1.6 × 10^−13^–1.0 × 10^−6^); the ratios between Boltzmann *k_B_T* and Josephson energies (defined as the normalized temperature *θ*) typically range in the (10^−4^–10^0^) interval. This usually corresponds to temperatures in the (10 mK–1 K) range [8,9,10,11]. The friction parameter, however, is subject to some speculation when the junction is biased on the zero-voltage step, and the work of [22] estimates that between 1 K and 100 mK, the “effective” dissipation could decrease about four orders of magnitude more. We will not here speculate further on the actual value of zero-voltage effective dissipation in real junctions, but simply consider the RCSJ model over a rather wide interval of the loss term in the Langevin equation in an effort to compare statistical models with the Langevin simulations.

## 4. Results for Thermal Initial Conditions

**Figure 2:**
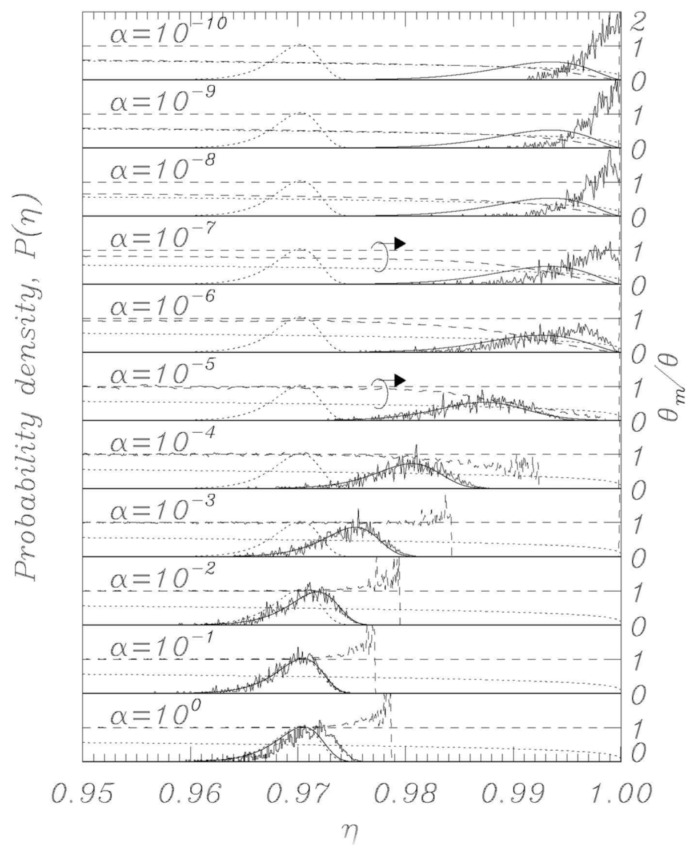
Numerically obtained escape distributions (jagged lines) along with Kramers (dotted) and BHL model (solid curve) for a sweep rate $\dot{\eta }=10^{-8}$. The BHL model closely follows the numerical results down to *α* = 10^−6^. Normalized temperature *θ* is 10^−3^. Also displayed are the measured temperature *θ_m_* (dashed curve), the thermodynamic temperature (dashed horizontal line), and the extreme low temperature model result for the sweep-induced temperature given by Equation (14) (dotted curve).

**Figure 3:**
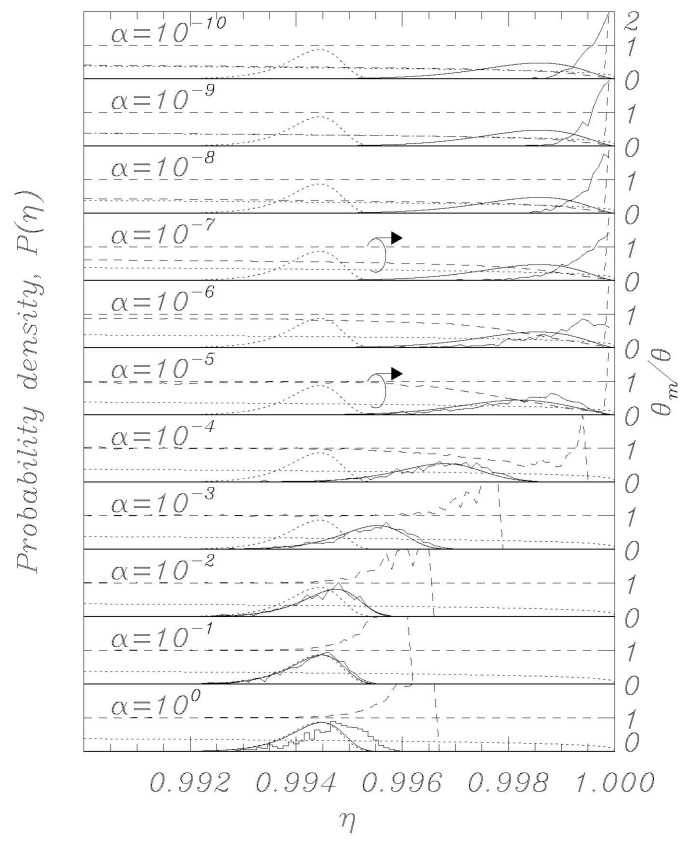
Same as in Figure 2, except for the normalized temperature *θ* = 10^−4^.

**Figure 4:**
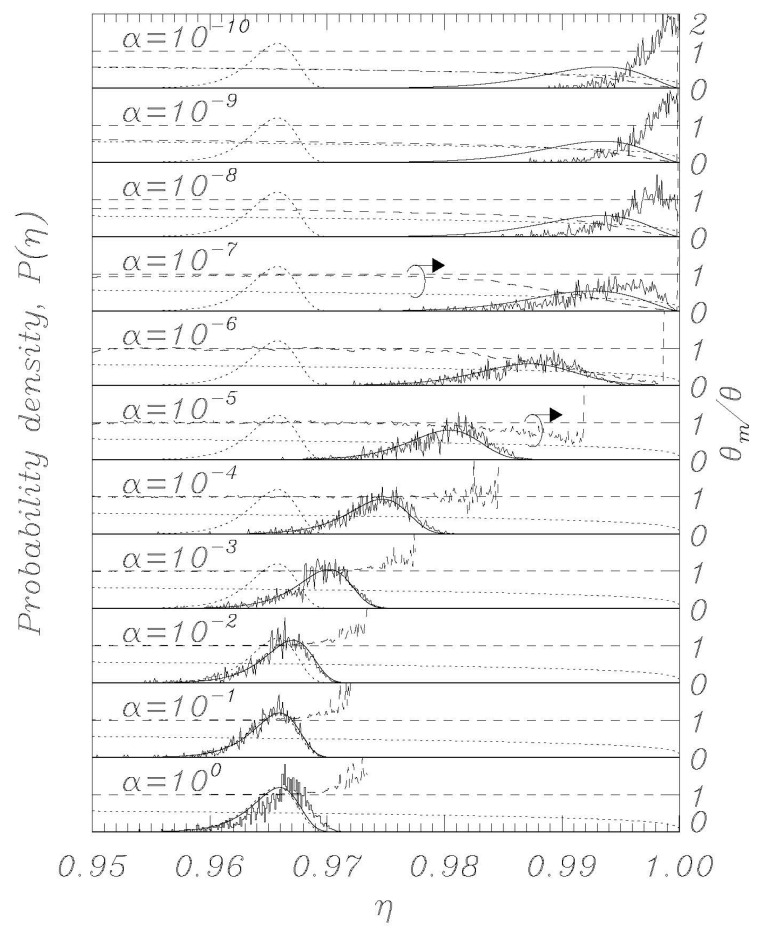
Same as in Figure 2, except for the normalized sweep rate $\dot{\eta }=10^{-9}$.

**Figure 5:**
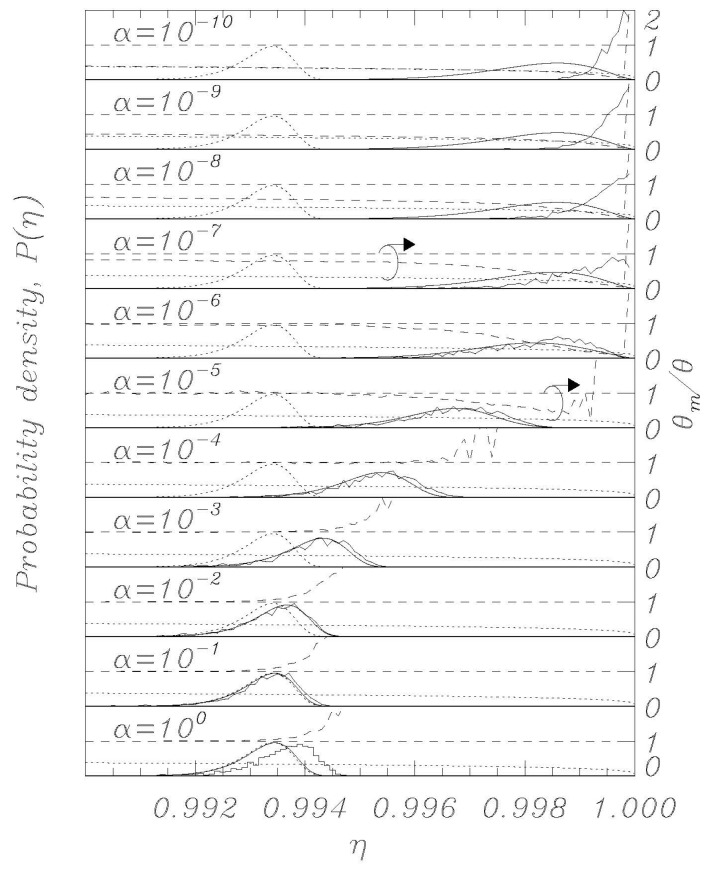
Same as in Figure 3, except for the normalized sweep rate $\dot{\eta }=10^{-9}$.

**Figure 6:**
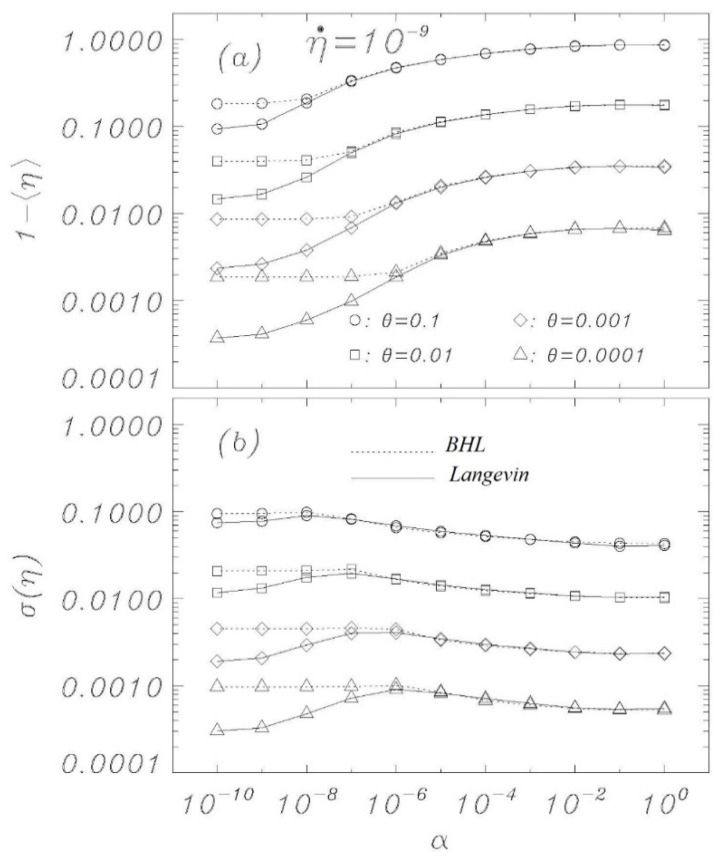
Mean (a) and standard deviation (b) of the escape current distributions for a variety of parameters given in the figures. Each Langevin marker represents the statistics of 10,000 escape simulations for $\dot{\eta }=10^{-9}$, initiated with thermal initial conditions at *η* = 0. Comparable data from the BHL model are also displayed.

Using the thermal initial conditions described above, representative distributions for different values of the dissipation parameter *α* are visualized in Figure 2, Figure 3, Figure 4, Figure 5 and Figure 6; the dotted curves (single peak distributions) in each subpanel of Figure 2, Figure 3, Figure 4 and Figure 5 show the results expected from Kramers’ statistical model (Equation (3)). Figure 2 and Figure 3 represent the same sweep rate *η* = 10^−8^ for two values of *θ*, 10^−3^ (Figure 2) and 10^−4^ (Figure 3). Figure 4 and Figure 5 represent a sweep rate *η* = 10^−9^ for *θ* = 10^−3^ (Figure 4) and *θ* = 10^−4^ (Figure 5).

When conducting bias sweep simulations, it is self-evident that the statistical accuracy at the right tail of the distribution corresponding to higher values of *η* will be poor, since very few events will reach those high values. In order to mitigate this effect, we have included in our simulations a feature to simulate those rare events through the following scheme. A large number, *N* = 1000, of independent simulations (an ideal gas) are initiated with different initial conditions according to the above description, and they proceed synchronously in *η* with different realizations of the stochastic noise. The *i*th simulation tracks the phase *φ_i_* (i = 1, 2,…, *N*) and has a statistical weight *w_i_* such that $\overset{N}{\underset{i=1}{\sum }}w_{i}=1$, where initially *w_i_* = *N*^−1^ for all *i*. When one of the phases, say *φ_i_*, records a switch, the corresponding *η* value is recorded with statistical weight *w_i_*. Another simulation, say *φ_j_*, is then randomly chosen, and we set $\left(\varphi_{i},{\dot{\varphi }}_{i}\right)=\left(\varphi_{j},\;{\dot{\varphi }}_{j}\right)$ and *w_i_* = *w_j_*/2, whereafter we set *w_j_* = *w_i_*. Then the *N* simulations again proceed synchronously until a simulation again records a switch. This procedure ensures that we always simulate *N* systems regardless of the actual statistical density of the system, and the statistical accuracy is therefore greatly enhanced for the rare events at the tail end of the distributions. The results of this rare-event algorithm can be seen in Figure 2, Figure 3, Figure 4 and Figure 5 as the solid histogram curves, which exhibit a near-uniform statistical uncertainty regardless of the observed density. Accompanying those curves are smooth solid curves which represent the BHL statistical model of the switching density. We observe that the agreement between the Langevin switching simulations and the BHL model is rather good for damping values *α* ≥ 10^−6^. For smaller friction values, the simulated distributions continue to approach *η* = 1 as friction is decreased, while the BHL model results become independent of friction for these extremely low friction values.

In order to explain the discrepancy between the BHL model and the Langevin simulations, we proceed to a linearization of Equation (1) around the potential minimum *φ_* = *sin*^−1^*η*, such that $\varphi =\varphi_{-}+\psi$, where |ψ|≪1 is the small-amplitude dynamical variable. The equation for ψ is:

(7) $$\ddot{\psi }+\alpha \dot{\psi }+\omega_{p}^{2}\;\psi =n\left(t\right)$$

with $\omega_{p}^{2}={cos}^{-1}\varphi_{-}$. This stochastic harmonic equation for ψ produces the two statistical equipartitioned moments:

(8) $$\omega_{p}^{2}<\psi^{2}>=<{\dot{\psi }}^{2}>=\theta$$

such that the average total normalized energy <*H*> for the small amplitude variable is given by

(9) $$<H>=\frac{1}{2}\omega_{p}^{2}<\psi^{2}>+\frac{1}{2}<{\dot{\psi }}^{2}>=\theta$$

Suppose now that we instantaneously change the bias current such that *ω_p_* changes by *dω_p_*. Given that the distributions ϱ(ψ) and $\varrho \left(\dot{\psi }\right)$ cannot change instantaneously, the instantaneous total energy change must therefore be given by

(10) $$d<H>=\omega_{p}<\psi^{2}>d\omega_{p}$$

Subsequent equipartition will produce the new equilibrium moment:

(11) $$<\psi^{2}>=\frac{<H>+d<H>}{\omega_{p}^{2}}$$

which, combined with Equation (10), gives:

(12) $$\frac{d<H>}{<H>}=\frac{d\omega_{p}}{\omega_{p}}=\frac{d\theta }{\theta }$$

(13) $$⟹\theta =\theta_{0}\frac{\sqrt[{4}]{1-\eta^{2}}}{\sqrt[{4}]{1-\eta_{0}^{2}}}$$

where we have *θ* = *θ*_0_ for *η* = *η*_0_. For *η*_0_ = 0, which we use in these simulations, we finally obtain

(14) $$\theta =\theta_{0}\sqrt[{4}]{1-\eta^{2}}$$

Thus, in the extremely low damping limit, where every change in *η* can be viewed as instantaneous compared to the equilibration time of the system, the sweep acts as an expanding confinement of an enclosed gas, which cools as the phase space expands. This notion is validated in Figure 2, Figure 3, Figure 4 and Figure 5, where we display the normalized temperatures of the ensemble of simulations as a function of the bias current. The measured normalized temperature of an ensemble is here given by the weighted average of the kinetic energy:

(15) $$\theta_{m}=\frac{\sum_{i=1}^{N}w_{i}{\dot{\varphi }}_{i}^{2}}{\sum_{i=1}^{N}w_{i}}$$

In the figures, in all the panels referring to *α* = 10^−5^ and *α* = 10^−7^, a circle encloses the lines referring to temperature data, and the arrows above the circles indicate that those data are to be referred to the vertical scale on the right. The horizontal dashed curve indicates the thermodynamic temperature set by the initial conditions and by the temperature in Equations (1) and (2). The dashed curve indicates the temperature *θ_m_* (Equation (15)) determined from the simulations, and the dotted curve indicates the result of Equation (14).

For the friction parameter above *α* = 10^−4^, we observe that the measured temperature mostly coincides with the thermodynamic temperature. Some positive deviations are observed at the tail end of the distributions, and these must be attributed to the fact that the inherent high energy of switching events dominates statistically when the entire system content is about to escape. As the friction is decreased, we observe the BHL cooling mechanism that depresses the measured temperature when switching begins to take place. This cooling reflects the fact that it takes time for the ensemble to replenish the statistical equilibrium of the remaining simulations when a high-energy simulation switches. For the extremely low friction simulations, we observe the cooling mechanism described in this work. The signature of this is that the system has cooled to the value given by Equation (14) prior to the switching events taking place, resulting in switching occurring at a significantly reduced temperature compared to the prescribed thermodynamic value. Consequently, the switching distribution is driven to higher *η* values compared to the ones predicted by the BHL model.

Figure 6 shows the condensation of the escape statistics into distribution peak position (Figure 6a) and width (Figure 6b) for $\dot{\eta }=10^{-9}$ and for different temperatures and damping parameters for both Langevin (solid interpolations) simulations and the BHL (dotted interpolations) model. The figure confirms the results from Figure 2, Figure 3, Figure 4 and Figure 5 but illuminates how the BHL model fails to account for the new cooling mechanism outlined in this paper for extremely low damping sweep experiments. Notice that the standard Kramers result is a constant at a value for high damping. Each marker for the Langevin simulations is constructed from 10,000 sweep simulations, starting at *η* = 0 with the thermal initial conditions given by the indicated thermodynamic temperature. The peak position is defined as the average of the 10,000 switching currents, while the width is calculated as the standard deviation of those values.

## 5. Conclusions

In a previous endeavor [20], the effect of the initial conditions on the escape from the washboard potential has been investigated systematically, revealing relevant properties of the process. Here, we have demonstrated that the model developed by Büttiker, Harris, and Landauer (BHL) [4] provides a satisfying account for the phenomenology of the RCSJ model of Josephson junctions as far as the escape from potential properties are concerned when dissipation is entered through a linear loss term in the equations, as long as the damping is not extremely small. The model captures, over wide parameter ranges, the most important features of the physical system traced by extensive numerical simulations of the dynamical equations performed imposing thermal initial conditions. We have reconfirmed that a relevant parameter that influences transitions between different dynamical conditions is the ratio *κ* between dissipation *α* and sweep rate $\dot{\eta }$. For this ratio below a certain threshold (i.e., for extremely low damping), the BHL model fails to reproduce the results of direct Langevin escape simulations, and we have developed a simple model of cooling that seemingly explains the phenomenon. We submit that the potential well acts as a confining cylinder for the trapped particle in the metastable state, and that the bias sweep acts as an expanding piston that effectively cools the gas. When the damping parameter is too small for the system to re-equilibrate during this cooling process, then the effective temperature remains below the thermodynamic expectation, and the escape therefore does not follow the expectations from either the Kramers or BHL models. Our result is in very good agreement with simulations for vanishing damping.

Here, as in the previous paper [20], we have seen that numerical simulations of the Langevin model provide very good agreement with theory, demonstrating that the features of the nonlinear RCSJ model can be approximated, with a high degree of accuracy, by statistical models, even for relatively low loss and temperatures. Significant deviations in experimental data from the classical model at low temperatures can be due to effects that critically depend on initial conditions or other perturbations during the experimental execution. The work described here, together with previous simulations of bias sweep protocols, suggests that the outcome of escape studies from bias sweep simulations are hypersensitive to perturbations when the zero-voltage damping becomes extremely low, which some indications point to for very low temperatures. A previous work [14] proposed the possibility that at very low temperatures, the low thermal conductivity of superconductive films could represent a problem for removing the heat generated in the junctions by the switches into the voltage state; a phenomenon which could limit the real temperature decrease in the junctions. It is not straightforward at this point to assess if (and how) the present work is directly related to experimental reality. Figure 2 and Figure 4 show that the low temperature “BHL cooling” begins for *θ* = 0.001 at values of the bias current close to those where the experiments depart from the Kramers model for a Josephson maximum critical current *I_c_* of the order of 2 μA (see, for example, [10,11]). This could be a coincidence and we point out that real experiments below a given temperature the position of the peaks of the statistical escape distributions tends to become independent on temperature and to occur always at the same value of the bias current, which is not what we see in the Langevin model here since going down in temperature the peaks keep moving toward higher bias currents (see Figure 3, Figure 5 and Figure 6a). However, we have demonstrated that the RCSJ Langevin simulations produce both a cooling mechanism consistent with the BHL model (in the range 10^−6^ < *α* < 10^−4^ for our simulations) and another cooling mechanism by the model of an expanding ideal gas that we presented in Section 4 for *α* < 10^−7^. Whether or not these interesting characteristics of the analyzed nonlinear model will have an impact on the behavior of real Josephson systems for very low temperature and dissipation is an issue to be investigated.

This project has been supported in part by the FEEL project (INFN, Italy).

## Figures and Tables

**Table 1:** Correspondence between BHL notation [4] and ours. Setting *m* = 1 means that the time normalization of BHL becomes the usual plasma frequency normalization that we employ.

Variable/Equation	BHL	This Paper
Noise in Langevin model	〈ξ(t)ξ(t′)〉=2γkTδ(t−t′)	Equation (2)
Josephson energy	$V_{0}$	$E_{j}$
Plasma free frequency	$\omega_{p}=\sqrt{\frac{V_{0}}{m}}$	$\omega_{0}$
Plasma frequency	ωA=ωp(1−F2V02)1/4	$\omega_{0}\omega_{p}=\omega_{0}{\left(1-\eta^{2}\right)}^{1/4}$
Normalized bias current	$\frac{F}{V_{0}}$	η
Characteristic time	$t_{0}=\frac{1}{\omega_{p}}$	$t_{0}=\frac{1}{\omega_{0}}$
Phase and derivatives	$\theta ,\;\;\dot{\theta }=\sqrt{\frac{V_{0}}{m}}\theta \prime ,\;\;\ddot{\theta }=\frac{V_{0}}{m}\theta \prime \prime$ $\dot{\theta }=\frac{d\theta }{dt},\;\theta \prime =\frac{d\theta }{d\tau },\;\;\tau =t/t_{0}$	$\varphi ,\;\dot{\varphi },\;\;\ddot{\varphi }$ $\dot{\varphi }=\frac{d\varphi }{d\tau }\;$
Normalized Equations	$\theta \prime \prime +G\theta \prime +sin\theta =\frac{F}{V_{0}}+\frac{\xi }{V_{0}}$ $\frac{V}{V_{0}}=\left(1-cos\theta \right)-\left(\frac{F}{V_{0}}\right)\theta$	$\ddot{\varphi }+\alpha \dot{\varphi }+sin\varphi =\eta +n$ $\frac{U}{E_{j}}=\left(1-cos\varphi \right)-\eta \varphi$
Normalized (1.2)	$\frac{\langle \xi \left(t\right)\xi \left(t\prime \right)\rangle }{V_{o}{}^{2}}=2G\frac{kT}{V_{0}}\delta \left(\tau -\tau \prime \right)$	〈n(τ)n(τ′)〉=2αθδ(τ−τ′)
Normalized thermal energy	$\frac{kT}{V_{0}}$	$\theta =\frac{k_{B}T}{E_{j}}$
Normalized damping	$G=\frac{\gamma }{\sqrt{mV_{0}}}$	α
Normalized noise	$\xi /V_{0}$	n
Barrier height	$E_{b}$	$E_{j}\Delta U$
BHL escape rate (Equation 3.11)	r=1+4αkTηIb−11+4αkTηIb+1(ηIbkT)(ωA2π)e−EbkT Ib=310I0[2(1−FV0)]5/4 $I_{0}=16\sqrt{mV_{0}}$ α: correction factor (~1)	$\Gamma =\frac{\sqrt{1+\frac{4k_{B}T}{{\alpha I}_{b}}}-1}{\sqrt{1+\frac{4k_{B}T}{{\alpha I}_{b}}}+1}\left(\frac{{\alpha I}_{b}}{k_{B}T}\right)\left(\frac{\omega_{p}\omega_{0}}{2\pi }\right)e^{-\frac{E_{j}\Delta U}{k_{B}T}}$ $I_{b}=\frac{3}{10}I_{0}{\left[2\left(1-\eta \right)\right]}^{5/4}$ $I_{0}=16E_{j}$ correction factor = 1

## Data Availability

Not applicable.

## References

[1] Kramers H.A. (1940). Brownian Motion in a Field of Force and the Diffusion Model of Chemical Reactions. H.A. Physica.

[2] van Duzer T., Turner C.W. (1999). Principles of Superconducting Devices and Circuits.

[3] Kurkijarvi J. (1972). Intrinsic Fluctuations in a Superconducting Ring Closed with a Josephson Junction. Phys. Rev. B.

[4] Büttiker M., Harris E.P., Landauer R. (1983). Thermal activation in extremely underdamped Josephson-junction circuits. Phys. Rev. B.

[5] Barone A., Cristiano R., Silvestrini P. (1985). Supercurrent Decay in Underdamped Josephson junctions: Nonstationary case. J. Appl. Phys..

[6] Silvestrini P., Liengme O.L., Gray K. (1988). Current Distributions of Thermal Switching in Extremely Underdamped Josephson Junctions. Phys. Rev. B.

[7] Caldeira A.O., Leggett A.J. (1981). Influence of Dissipation on Quantum Tunneling in Macroscopic Systems. Phys. Rev. Lett..

[8] Voss R.F., Webb R.A. (1981). Macroscopic Quantum Tunneling in 1-μm Nb Josephson Junctions. Phys. Rev. Lett..

[9] Washburn S., Webb R.A., Voss R.F., Faris S.M. (1985). Effects of Dissipation and Temperature on Macroscopic Quantum Tunneling. Phys. Rev. Lett..

[10] Yu H.F., Zhu X.B., Peng Z.H., Cao W.H., Cui D.J., Tian Y., Chen G.H., Zheng D.N., Jing X.N., Lu L. (2010). Quantum and Classical Resonant Escapes of a Strongly Driven Josephson Junction. Phys. Rev. B.

[11] Oelsner G., Revin L.S., Il’ichev E., Pankratov A.L., Meyer H.-G., Grönberg L., Hassel J., Kuzmin L.S. (2013). Underdamped Josephson Junction as a Switching Current Detector. Appl. Phys. Lett..

[12] Martinis J.M., Grabert H. (1988). Thermal Enhancement of Macroscopic Quantum tunneling: Derivation from Noise Theory. Phys. Rev. B.

[13] Clarke J., Wilhelm F.K. (2008). Superconducting Quantum Bits. Nature.

[14] Blackburn J.A., Cirillo M., Grønbech-Jensen N. (2016). A Survey of Classical and Quantum Interpretations of Experiments on Josephson Junctions at Very Low Temperatures. Phys. Rep..

[15] Grønbech-Jensen N., Cirillo M. (2005). Rabi-Type Oscillations in a Classical Josephson Junction. Phys. Rev. Lett..

[16] Marchese J.M., Cirillo M., Grønbech-Jensen N. (2007). Classical analogs for Rabi-oscillations, Ramsey-fringes, and spin-echo in Josephson junctions. Eur. Phys. J. Spec. Top..

[17] Grønbech-Jensen N., Castellano M.G., Chiarello F., Cirillo M., Cosmelli C., Merlo V., Russo R., Torrioli G., Ruggiero B., Delsing P., Granata C., Pashkin Y., Silvestrini P. (2006). Anomalous Thermal Escape in Josephson Junctions Perturbed by Microwaves. Quantum Computation in Solid State Systems.

[18] Silvestrini P., Palmieri V.G., Ruggiero B., Russo M. (1997). Observation of Energy Levels Quantization in Underdamped Josephson Junctions above the Classical-Quantum Regime Crossover Temperature. Phys. Rev. Lett..

[19] Cheng C., Cirillo M., Salina G., Grønbech-Jensen N. (2018). Nonequilibrium Transient Phenomena in the Washboard Potential. Phys. Rev. E.

[20] Cheng C., Salina G., Grønbech-Jensen N., Blackburn J.A., Lucci M., Cirillo M. (2020). Modeling Escape from a One-Dimensional Potential Well at Zero or Very Low Temperatures. J. Appl. Phys..

[21] Jensen L.F.G., Grønbech-Jensen N. (2019). Accurate Configurational and Kinetic Statistics in Discrete-Time Langevin Systems. Mol. Phys..

[22] Silvestrini P., Pagano S., Cristiano R., Liengme O., Gray K.E. (1988). Effect of Dissipation on Thermal Activation in an Underdamped Josephson Junction: First Evidence of a Transition between Different Damping Regimes. Phys. Rev. Lett..

